# Risk Interval of Immune Thrombocytopenia in Children After Influenza Infection

**DOI:** 10.1111/ped.70582

**Published:** 2026-10-13

**Authors:** Kyosuke Ibi, Yoshihisa Miyamoto, Motohiro Kato, Hideo Yasunaga

**Affiliations:** ^1^ Department of Pediatrics, Graduate School of Medicine The University of Tokyo Tokyo Japan; ^2^ Department of Clinical Epidemiology and Health Economics, Graduate School of Medicine The University of Tokyo Tokyo Japan; ^3^ Department of Real‐World Evidence, Graduate School of Medicine The University of Tokyo Tokyo Japan

**Keywords:** epidemiology, immune thrombocytopenia, immune thrombocytopenic purpura, immunology, influenza

## Abstract

**Background:**

The temporal association between influenza and immune thrombocytopenia (ITP) is poorly characterized. This study examined the risk interval between influenza infection and newly diagnosed ITP in children.

**Methods:**

We enrolled individuals aged 1–15 years newly diagnosed with ITP between 2005 and 2024, using the Japanese large‐scale administrative JMDC Database. We conducted a nested case–control study and self‐controlled case series to evaluate the risk of ITP within 28 days (categorized into 7‐day intervals) of influenza diagnosis. We estimated the risk interval from influenza diagnosis to ITP onset using conditional logistic and Poisson regressions.

**Results:**

We identified 2255 individuals diagnosed with ITP, of whom 1324 met the eligibility criteria. In the nested case–control analysis, the adjusted odds ratio of preceding influenza diagnosis was the highest within 1–7 days before ITP onset (3.13; 95% confidence interval 1.38–7.12) compared with the matched controls. In the self‐controlled case series, the incidence rate ratio for ITP was the highest within 1–7 days after influenza diagnosis compared to the baseline period (2.44; 95% confidence interval 1.16–5.14). In the sensitivity and post hoc analyses for different definitions of influenza diagnosis and ITP onset dates, ITP onset peaked within 1–14 days of influenza diagnosis.

**Conclusions:**

The risk of ITP appeared to be highest during the first 1–2 weeks after influenza diagnosis, particularly around the first week.

## 1 Introduction

Immune thrombocytopenia (ITP) is an acquired autoimmune disorder characterized by a low platelet count resulting from platelet destruction and impaired platelet production [1]. Classically, a preceding infection is reported in approximately two‐thirds of newly diagnosed ITP cases in children. Virus‐associated ITP is classically reported to develop approximately 1–4 weeks after a preceding infection [2].

Influenza is a representative upper respiratory tract infection that can trigger ITP [2, 3, 4]. Although considerable attention has been paid to ITP following influenza vaccination, studies on the clinical impact of influenza infection as a trigger of ITP are lacking [5, 6]. Consequently, the strength and temporal nature of ITP following influenza infection remain largely unexplored. Given the high seasonal prevalence and significant public health burden of influenza [7], it is crucial to clarify the temporal association between influenza and ITP. Previous studies on the risk interval between influenza and ITP were beset by methodological insufficiencies. For example, previous studies on the association between influenza infection and ITP were limited to case reports and did not estimate the magnitude of the risk for ITP relative to non‐infectious individuals [3, 4]. Even for ITP following viral infections other than influenza, previous retrospective studies have estimated the risk interval (e.g., the timing of infection) through patient interviews or serological testing performed after the diagnosis of ITP [8, 9]. Interview‐based assessments may be susceptible to recall bias [10], whereas post‐diagnosis serological testing may have limited temporal resolution for identifying the precise timing of infection onset, potentially leading to misclassification of the risk intervals [11]. Consequently, data on the temporal relationship between influenza infection and subsequent ITP onset are lacking.

Therefore, we aimed to examine the risk interval between influenza infection and ITP onset. We conducted a nested case–control study and self‐controlled case series (SCCS) using data from a large administrative claims database in Japan.

## 2 Methods

### 2.1 Study Design

We performed a nested case–control study and an SCCS using a commercially available administrative claims database from JMDC Inc. (Tokyo, Japan) [12]. This retrospective study involving human participants was conducted in accordance with the ethical standards of the institutional and national research committee and with the 1964 Helsinki Declaration and its later amendments or comparable ethical standards. The institutional Review Board of The University of Tokyo approved this study (approval number: 10862–(6) [May 12, 2026]). All statistical analyses were conducted using Stata/SE (version 18.0; StataCorp, College Station, Texas, USA).

### 2.2 Data Source

The JMDC database includes health insurance claims data on outpatients, inpatients, and pharmacies for the employees of medium‐to large‐scale companies and their family members since 2005. The observed cumulative population is 23 million. This database contains demographic data, such as year and month of birth, sex, and medical and pharmacy claims as follows: names and dates of medical practice; diagnosis based on the International Classification of Diseases, 10th Revision (ICD‐10) codes, Japanese procedure codes and diagnosis dates; drug names based on World Health Organization Anatomical Therapeutic Chemical codes, dosages, and prescription dates; and claims type, such as inpatient and outpatient [13]. Definitions of the ICD‐10 codes, Japanese procedure codes, and Anatomical Therapeutic Chemical codes used in the research are presented in Table S1.

### 2.3 Source Population and Outcome Definition

The source population was individuals aged 1–15 years registered in the database from January 1, 2005, to July 31, 2024. We defined the start of the observation period by the date of database registration or the individual's first birthday, whichever occurred later. The end of the observation period was defined by disenrollment from insurance, the individual's 16th birthday, or the end of the data availability period, whichever occurred first. The primary outcome was ITP diagnosis, as defined by ICD‐10 codes. We did not include individuals diagnosed with secondary thrombocytopenia (including drug‐induced or heparin‐induced thrombocytopenia and pseudothrombocytopenia) or other non‐thrombocytopenic purpura (including thrombotic thrombocytopenic purpura and IgA vasculitis). First, we identified individuals diagnosed with ITP during the observational period. We defined the date of ITP onset (index date) as the first complete blood count test within the month of ITP diagnosis, which was recorded using Japanese procedure codes. We excluded individuals without a complete blood count test within the month of ITP diagnosis because the test is a prerequisite for ITP diagnosis. The exact date of ITP was unavailable because the diagnostic codes in the JMDC database were recorded monthly. Therefore, the date of the relevant diagnostic examination performed within the diagnosis month was used as a proxy for the ITP diagnosis date. The study population consisted of individuals who were not diagnosed with ITP within a 6‐month lookback period before the index date. We did not include individuals registered for less than 6 months before the index date to confirm the absence of prior ITP diagnosis and to evaluate covariates with the lookback period.

### 2.4 Definition of Exposure

We designated the diagnosis of influenza as an exposure. The exposure was extracted using a combination of ICD‐10 diagnostic codes and diagnostic tests recorded via the Japanese procedure codes. The exposure date was defined as the date of the first diagnostic test for influenza within the same month as the influenza diagnosis. Influenza may have occurred several times during the study period. If multiple exposures occurred within 14 days, the earliest exposure date was considered [14].

### 2.5 Nested Case–Control Design

From the study population, we randomly selected ten controls for each case on the index date. We chose individuals without ITP and those with other diseases with thrombocytopenia (excluding diseases) in the month of the index date as controls. The cases and controls were matched by age (same month and year of birth), sex, and time of registration in the database (same month and year of registration) using risk‐set sampling with replacement [15]. The risk interval was set at 28 days before the index date, based on the risk interval in previous studies [2, 3, 4]. We categorized cases and controls according to whether they experienced the exposure within (i) 1–7 days, (ii) 8–14 days, (iii) 15–21 days, (iv) 22–28 days, and (v) no infection within 28 days preceding the index date. Conditional logistic regression analyses were used to estimate the odds ratios (ORs) and 95% confidence intervals (CIs) for each interval, using (v) as a reference. We adjusted for the following variables: age, sex, time of registration in the database (same month and year of registration), number of categories in the Pediatric Complex Chronic Condition System version [3, 16] number of clinic visits, number of drugs prescribed, and history of hospitalization in the pediatric department during the 6‐month lookback period.

### 2.6 SCCS Design

We used an SCCS design to calculate the incidence rate ratio (IRR) of ITP in the temporal risk interval following the influenza diagnosis in individuals diagnosed with ITP. The SCCS design compares the intra‐person incidence rates of outcomes across different study intervals with respect to the timing of exposure [17, 18]. This study design has been used for investigating the relationship of an intermittent exposure with transient effects on acute events. The advantage of SCCS lies in its implicit ability to adjust for time‐invariant confounders, such as genetic or socioeconomic factors. Our study benefited from the SCCS design because genetic and socioeconomic conditions are among the risk factors for ITP [19, 20].

The SCCS design makes the following three key assumptions for providing valid and unbiased estimates [18]. First, the occurrence of an event should not (appreciably) affect the subsequent exposures. To meet this assumption, a washout period (exposure date and pre‐exposure period: 1–14 days before the exposure date) was established because screening tests for influenza may sometimes be performed on or after the date of an outpatient visit with an ITP presentation. Second, the event rates are constant within the intervals. We explicitly adjusted for age and season as time‐varying confounding factors in the statistical model [21, 22, 23]. Third, the events must be independently recurrent or rare. To satisfy this assumption, we limited the events to the initial ITP diagnosis. As illustrated in Figure 1, we divided the entire observation period into seven parts according to the exposure date: (i) pre‐exposure period, 1–14 days before the exposure date; (ii) exposure date (day 0); (iii) risk period 1, 1–7 days; (iv) risk period 2, 8–14 days; (v) risk period 3, 15–21 days; and (vi) risk period 4, 22–28 days after the exposure date. The entire observation period, excluding intervals (i)–(vi), was defined as (vii) the baseline period. The number of individuals experiencing events within each interval was counted. Under the SCCS framework, individuals with ITP who did not experience exposure (influenza diagnosis) during the observation period were included in the model. Although they do not directly contribute to the estimation of exposure‐associated IRRs, they do contribute information to the estimation of time‐varying confounding factors [1, 24]. We conducted conditional Poisson regression analyses to estimate the IRRs for ITP diagnosis in each risk period compared to the baseline period. The logarithm of the interval length (person‐time) was included as an offset term. We adjusted for age bands (1–4, 5–8, 9–12, and 13–15 years) and seasons in 3‐month blocks (March–May; June–August; September–November; and December, January, and February).

**FIGURE 1:**
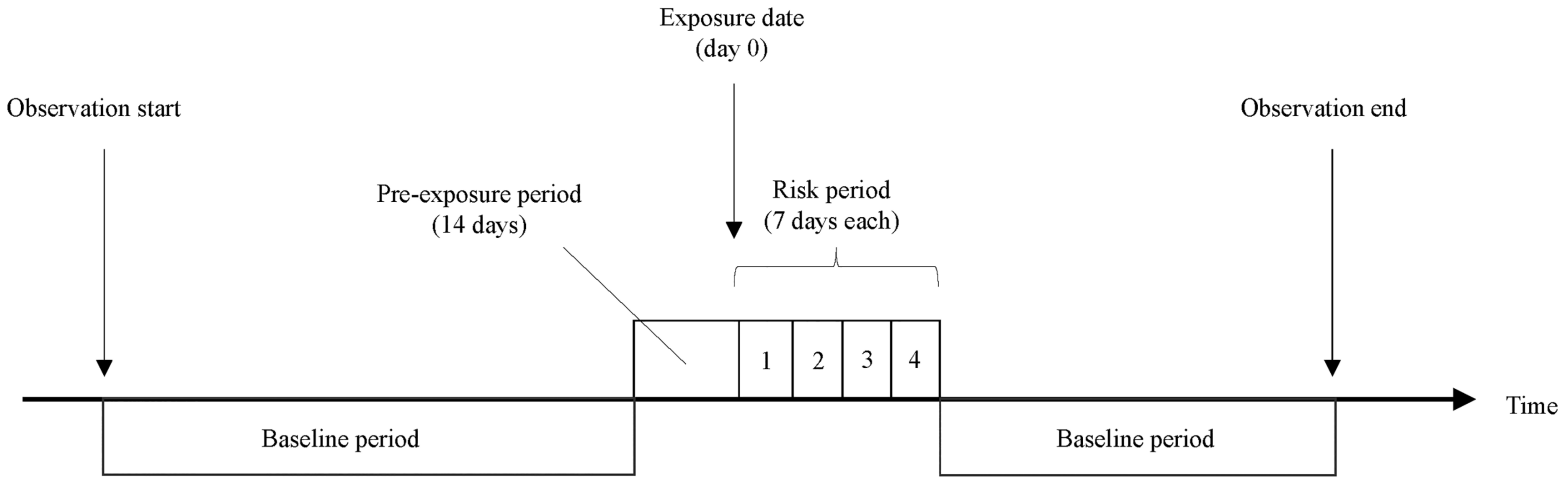
Diagram of the observation period in the self‐controlled case series design.

### 2.7 Sensitivity Analysis

We conducted three sensitivity analyses to enhance the robustness of our findings. We sought to manage the uncertainty of the ITP onset date and improve the specificity of the influenza diagnosis and its date, because misclassification may be a concern in claims databases. In sensitivity analysis 1, we first focused on patients hospitalized with ITP within a month of ITP diagnosis to enhance the specificity of ITP diagnosis [25]. The ITP onset date was defined as the date of hospitalization for ITP. Then, we further focused on patients administered ITP‐specific treatment (intravenous or oral corticosteroids or intravenous immunoglobulin) within the month of ITP diagnosis [1]. The ITP onset date was defined as the date of the first use of ITP‐specific treatment. In sensitivity analysis 2, influenza was defined by adding prescriptions for anti‐influenza drugs to the diagnostic and testing codes within the same month to increase the specificity of influenza diagnosis. In sensitivity analysis 3, the results were verified by defining independent influenza episodes at 7‐ and 30–day intervals.

### 2.8 Post Hoc Analysis

We conducted an exploratory analysis using a narrow risk interval to facilitate a more detailed investigation of the timing of ITP onset immediately after influenza diagnosis. In the post hoc nested case–control analysis, we categorized both cases and controls according to whether they experienced influenza diagnosis within (i) 1–3 days, (ii) 4–7 days, (iii) 8–10 days, (iv) 11–14 days, (v) 15–21 days, (vi) 22–28 days, or (vii) no infection within 28 days before the index date. In the post hoc SCCS analysis, we partitioned the whole observation period into nine parts with respect to the exposure date: (i) pre‐exposure period: 1–14 days before the exposure date, (ii) exposure date (day 0), (iii) risk period 1: 1–3 days, (iv) risk period 2: 4–7 days, (v) risk period 3: 8–10 days, (vi) risk period 4: 11–14 days, (vii) risk period 5: 15–21 days, (viii) risk period 6: 22–28 days after the exposure date, and (ix) baseline period.

## 3 Results

We first identified 2255 individuals diagnosed with ITP between the ages of 1 and 15 years (Figure 2). After applying the exclusion criteria, 931 individuals were excluded, leaving 1324 eligible individuals newly diagnosed with ITP during the study period. Among the identified cohort, 948 patients were coded as D693 (immune thrombocytopenia) and 376 as D694 (other primary thrombocytopenia). Of the 376 patients with D694, 352 (93.6%) had the subcode D694‐4 (thrombocytopenic purpura).

**FIGURE 2:**
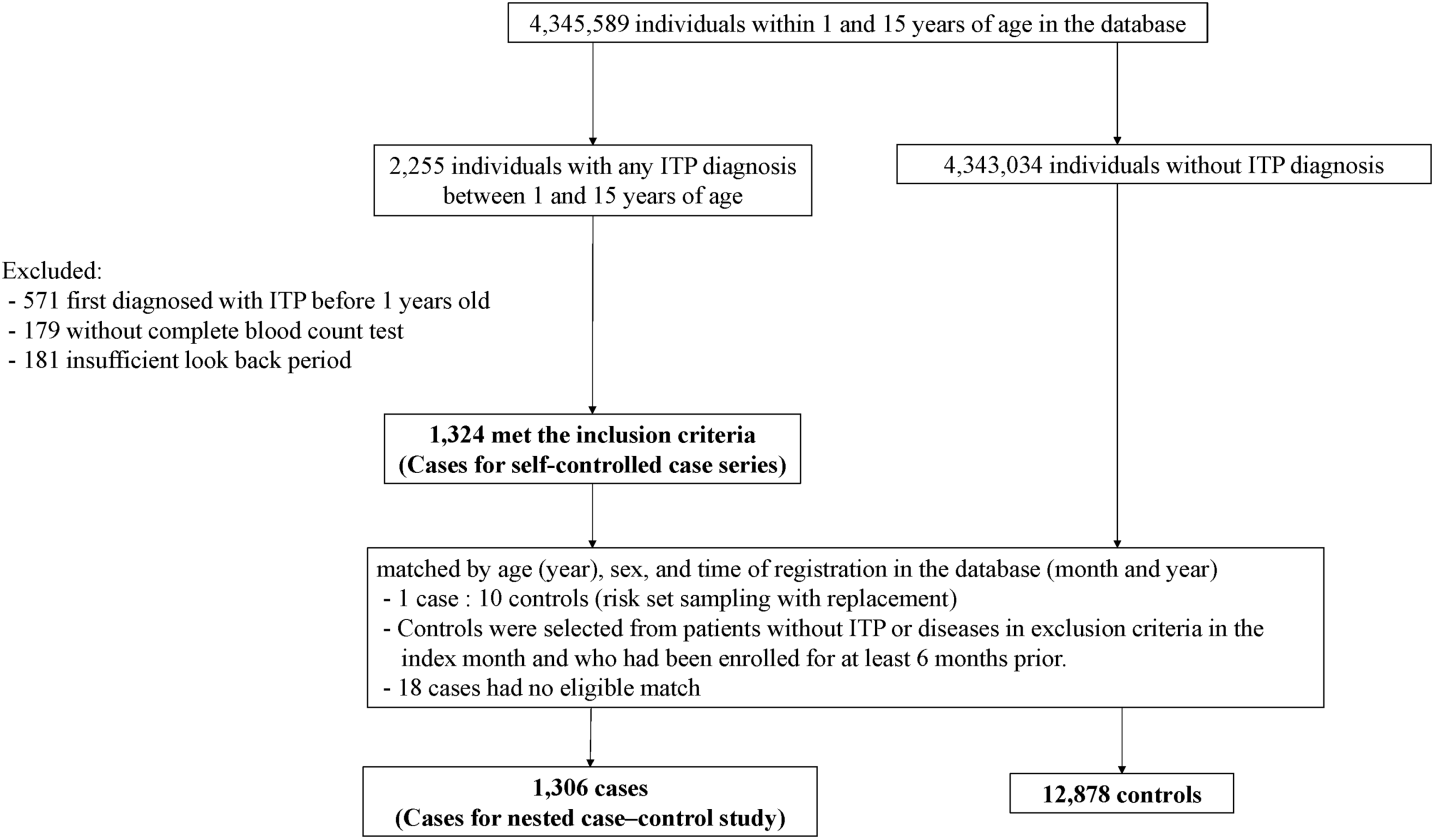
Patient flowchart. ITP, immune thrombocytopenia.

### 3.1 Nested Case–Control Study

We matched 1306 individuals with ITP to 12,878 individuals without ITP, and excluded 18 individuals with ITP who were not matched. The median age and male proportion of the matched participants were 4.9 (interquartile range: 2.6–9.7) years and 53.0%, respectively (Table 1). The case cohort had more categories in the Pediatric Complex Chronic Condition System, version 3, and a greater number of hospital visits, medications during the lookback period, and hospitalizations to the pediatric department than those in the control cohort. ITP was most prevalent in spring and summer, and least prevalent in winter.

**TABLE 1:** Characteristics of patients in the nested case–control design.

	Controls (*n* = 12,878)	Cases (*n* = 1306)	Standard mean difference
Age, years	4.8 (2.6–9.6)	4.9 (2.6–9.7)	< 0.001
Male	6838 (53.1%)	692 (53.0%)	< 0.001
Pediatric complex chronic condition system version 3			
Neurological	135 (1.0%)	37 (2.8%)	0.16
Cardiovascular	90 (0.7%)	32 (2.5%)	0.19
Respiratory	49 (0.4%)	14 (1.1%)	0.10
Renal and urologic	38 (0.3%)	12 (0.9%)	0.11
Gastrointestinal	28 (0.2%)	20 (1.5%)	0.23
Hematologic or immunologic	105 (0.8%)	75 (5.7%)	0.44
Metabolic	89 (0.7%)	34 (2.6%)	0.21
Other congenital or genetic defect	108 (0.8%)	32 (2.5%)	0.16
Malignancy	77 (0.6%)	38 (2.9%)	0.26
Premature and neonatal	42 (0.3%)	11 (0.8%)	0.08
Device and technology usage	16 (0.1%)	6 (0.5%)	0.09
Transplant	4 (0.03%)	6 (0.5%)	0.16
Number of hospital visits	4 (2–9)	6 (3–10)	0.21
Number of medicines	8 (3–14)	11 (5–17)	0.37
Hospital admissions	44 (0.3%)	18 (1.4%)	0.16
Year of onset			< 0.001
2009	40 (0.3%)	4 (0.3%)	
2010	120 (0.9%)	12 (0.9%)	
2011	114 (0.9%)	12 (0.9%)	
2012	282 (2.2%)	29 (2.2%)	
2013	287 (2.2%)	31 (2.4%)	
2014	493 (3.8%)	51 (3.9%)	
2015	631 (4.9%)	64 (4.9%)	
2016	880 (6.8%)	91 (7.0%)	
2017	1001 (7.8%)	101 (7.7%)	
2018	1156 (9.0%)	117 (9.0%)	
2019	1572 (12.2%)	158 (12.1%)	
2020	1228 (9.5%)	123 (9.4%)	
2021	1086 (8.4%)	110 (8.4%)	
2022	1191 (9.2%)	121 (9.3%)	
2023	1763 (13.7%)	178 (13.6%)	
2024	1034 (8.0%)	104 (8.0%)	
Season			< 0.001
Spring	3526 (27.4%)	357 (27.3%)	
Summer	3496 (27.1%)	353 (27.0%)	
Fall	3038 (23.6%)	311 (23.8%)	
Winter	2818 (21.9%)	285 (21.8%)	

*Note:* Data are presented as *n* (%) or median (interquartile range).

After adjusting for the measured confounders, individuals with ITP were found to have a greater exposure to influenza diagnosis than their matched controls. The adjusted ORs were elevated within 1–7 days before the ITP onset (adjusted OR, 3.13; 95% CI, 1.38–7.12; Figure 3A). The risk declined from the 8th day onward.

**FIGURE 3:**
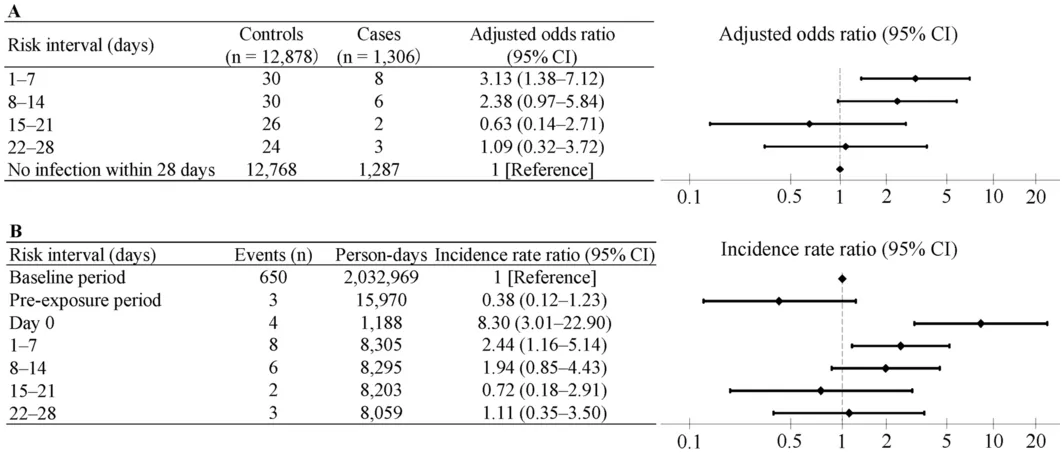
Risk interval from influenza to the onset of immune thrombocytopenia (A: Nested case–control study, B: Self‐controlled case series). (A) Conditional logistic regression was performed to estimate the odds ratios comparing influenza episodes among cases and controls after adjusting for each category viz. age, sex, time of registration in the database, each category of Pediatric Complex Chronic Condition system version 3, number of hospital visits, number of medicines prescribed, and history of admission to the pediatric department. (B) The incidence rate ratio was estimated using a conditional Poisson regression and adjusted for age band and seasonal effects. CI, confidence interval.

In sensitivity analysis 1, which focused on individuals hospitalized with ITP and those administered ITP‐specific treatment, we identified 915 cases and 9043 controls, and 813 cases and 8034 controls, respectively. The adjusted OR peaked within 1–7 days before the ITP onset (adjusted OR, 3.57 and 5.00; 95% CI, 1.12–11.36 and 1.48–16.91; Tables S2A and S3A). The point estimates of the adjusted ORs in sensitivity analyses 2 and 3 also peaked within 1–7 days before ITP onset, showing a trend similar to that of the main analysis (Tables S4A–S6A). According to the post hoc analysis, the adjusted ORs were elevated within 4–7 days before the ITP onset (adjusted OR, 3.35; 95% CI, 1.31–8.61) and 8–10 days before the ITP onset (adjusted OR, 6.38; 95% CI 1.84–22.07), while ITP occurred in only six and four cases during the interval (Table S7A).

### 3.2 SCCS

All 1324 individuals with ITP were included in the SCCS analysis. A total of 676 individuals experienced at least one episode of influenza infection. The point estimate of the IRR was lower during the pre‐exposure period compared with baseline (IRR, 0.38; 95% CI, 0.12–1.23; Figure 3B). The highest risk was observed on the date of influenza diagnosis (IRR, 8.30; 95% CI, 3.01–22.90). During the risk intervals, the IRR peaked within 1–7 days after the influenza diagnosis (IRR, 2.44; 95% CI, 1.16–5.14).

Among the 915 individuals hospitalized for ITP and 813 individuals administered ITP‐specific treatment in sensitivity analysis 1, a total of 427 and 382 had experienced at least one episode of influenza, respectively. The point estimates of the IRR for individuals hospitalized for ITP were similar to those of the main analysis, peaking within 1–7 days after the influenza diagnosis during the risk intervals (IRR, 2.04; 95% CI, 0.72–5.75; Table S2B). The point estimates for individuals administered ITP‐specific treatment peaked within 8–14 days after the influenza diagnosis during the risk intervals (IRR, 2.42; 95% CI, 0.88–6.69; Table S3B). In sensitivity analysis 2, a total of 646 individuals with at least one episode of influenza infection were administered anti‐influenza drugs. The point estimates of the IRR in sensitivity analyses 2 and 3 also showed a similar trend to those of the main analysis, as shown in Tables S4B–S6B. In the post hoc analysis, the risk for ITP was concentrated within 4–7 days and 8–10 days after the influenza diagnosis (IRR, 3.25; 95% CI, 1.40–7.54, and IRR, 3.01; 95% CI, 1.11–8.20, respectively) (Table S7B).

## 4 Discussion

Using a large Japanese claims database, we investigated the temporal association between influenza infection and newly diagnosed ITP in children. Our nested case–control study and SCCS yielded consistent findings, i.e., ITP following influenza peaked within 7 days after influenza diagnosis in the main analysis. In the post hoc analysis, the occurrence of ITP did not increase soon after influenza diagnosis and was concentrated within 4–10 days thereafter. A major strength of this study is the epidemiological evidence of the risk interval between influenza and ITP onset.

In our study, the risk of ITP rose sharply within the interval immediately after the diagnosis of influenza. These findings align with the latent period of ITP following influenza infection reported in previous case reports, wherein ITP occurred 3–4 days after influenza symptoms [3, 4]. The slight gap in the risk interval from previous studies could be related to the definition of influenza onset. The classification of influenza was diagnosis‐based in our study and symptom‐based in previous case reports. In some individuals, there may be a lag between the onset of influenza symptoms and diagnosis. In general, the interval between influenza symptoms and diagnosis is relatively short, with most patients being evaluated within 1–2 days of symptom onset [26]. Consequently, the true risk window relative to the onset of biological infection may begin slightly earlier than the observed window relative to the diagnosis date. Overall, across the multiple sensitivity and post hoc analyses, the peak risk of ITP clustered broadly within 1–14 days following influenza diagnosis, confirming the robustness of our temporal findings, regardless of the analytical approach or definition of the onset date. The differences within analyses warrant cautious interpretation, especially in post hoc analysis, in which the case count per interval decreased owing to shorter risk windows. However, the overall incidence of ITP appeared to be highest during the first 1–2 weeks after influenza diagnosis. The point estimate was attenuated toward the null when using a more specific definition for influenza diagnosis in sensitivity analysis 2, with the overall risk pattern remaining materially unchanged.

The mechanisms underlying ITP following influenza infection are not well understood. However, our results align with a presumed mechanism involving a rapid and non‐specific cascade of platelet destruction in ITP following influenza infection [27, 28, 29].

The observed risk of ITP on the date of influenza diagnosis may reflect the influence of biases, such as detection bias or reverse causation, rather than the true effect. First, the exclusively high risk of ITP on the date of influenza diagnosis may stem from detection bias or coding practices because individuals admitted to the hospital with ITP underwent influenza screening tests as part of the hospital's infection control measures [30]. This may be supported by the following findings: (1) the point estimate of IRRs on the date of influenza diagnosis was higher in sensitivity analysis 1, and (2) no cases were observed on the exposure date in sensitivity analysis 2. Second, subclinical ITP was incidentally detected during a visit for the infection, and screening for influenza was performed during a visit for ITP symptoms (detection bias) [31]. The decreased point estimate of IRRs during the pre‐exposure period may be explained by the outcome–exposure association; that is, individuals diagnosed with ITP had less exposure to infected individuals with influenza during acute ITP management.

This study has limitations inherent to its claims database origin. First, the risk interval for ITP following the date of influenza diagnosis may have been misclassified because ITP onset was defined based on the first complete blood count test in the main analysis. If blood tests had been performed for other reasons in the same month as the ITP diagnosis, the estimated diagnosis date may have been earlier than the date of ITP onset. However, the results of sensitivity analysis 1, in which the ITP onset date was defined as the date of hospitalization and administration of ITP‐specific treatment, were broadly compatible with those of the main analysis. This effect can be considered negligible. Second, because data on vaccines were not available in the claims database, the extent to which vaccine‐induced ITP was included was unclear. Because vaccines are typically administered when individuals are not infected with influenza, the effect of vaccination would be to reduce the association between influenza infection and ITP.

In conclusion, the risk of ITP appeared to be the highest during the first 1–2 weeks after influenza diagnosis, particularly during the first week. Our data identifying the high‐risk periods will provide general clinicians with information regarding the presentation of ITP following influenza infection.

## Author Contributions

K.I., Y.M., and H.Y. designed the study; K.I. and Y.M. collected and analyzed the data and wrote the manuscript; M.K. and H.Y. provided technical support and conceptual advice and revised the manuscript. All the authors have read and approved the final version of this manuscript.

## Funding

This work was supported by grants conferred by the Ministry of Health, Labour and Welfare, Japan (26AA2006).

## Consent

The requirement for informed consent was waived because the analysis was restricted to anonymized data.

## Conflicts of Interest

Y.M. has an academic affiliation with the Department of Real‐World Evidence, which is a cooperative program between the University of Tokyo and DeSC Healthcare. K.I., M.K., and H.Y. have no conflicts of interest to disclose.

## Supporting information


**Table S1:** Definitions of International Classification of Diseases and Related Health Problems 10th Revision codes, Japanese procedure codes, and Anatomical Therapeutic Chemical codes.
**Table S2:** Sensitivity analysis 1: Risk interval from influenza to hospitalization for immune thrombocytopenia.
**Table S3:** Sensitivity analysis 1: Risk interval from influenza to the administration of immune thrombocytopenia treatment.
**Table S4:** Sensitivity analysis 2: Risk interval from influenza treatment with anti‐influenza medication to the onset of immune thrombocytopenia.
**Table S5:** Sensitivity analysis 3: Risk interval from influenza to the onset of immune thrombocytopenia, considering influenza episodes more than 7 days apart separately.
**Table S6:** Sensitivity analysis 3: Risk interval from influenza to the onset of immune thrombocytopenia, considering influenza episode more than 30 days apart separately.
**Table S7:** Post hoc analysis: Risk interval from influenza to the onset of immune thrombocytopenia with narrow risk intervals.

## Data Availability

The datasets analyzed during the current study are not publicly available due to contracts with the hospitals providing data to the database.
