# Supplementary material for: Risk Interval of Immune Thrombocytopenia in Children After Influenza Infection
Source: Pediatr Int. 2026 Oct 13;68(1):e70582. doi: 10.1111/ped.70582 (PMC13659412; doi:10.1111/ped.70582)
Supplement: Supplementary file 1 [file PED-68-e70582-s001.docx]

**Supporting information**

This file contains supplementary tables for the article titled Risk interval of immune thrombocytopenia in children after influenza infection.

Index

**Table S1.** Definitions of International Classification of Diseases and Related Health Problems 10th Revision codes, Japanese procedure codes, and Anatomical Therapeutic Chemical codes

**Table S2.** Sensitivity analysis 1: Risk interval from influenza to hospitalization for immune thrombocytopenia

A: Nested case–control study

B: Self-controlled case series

**Table S3.** Sensitivity analysis 1: Risk interval from influenza to the administration of immune thrombocytopenia treatment

A: Nested case–control study

B: Self-controlled case series

**Table S4.** Sensitivity analysis 2: Risk interval from influenza treatment with anti-influenza medication to the onset of immune thrombocytopenia

A: Nested case–control study

B: Self-controlled case series

**Table S5.** Sensitivity analysis 3: Risk interval from influenza to the onset of immune thrombocytopenia, considering influenza episodes more than 7 days apart separately

A: Nested case–control study

B: Self-controlled case series

**Table S6.** Sensitivity analysis 3: Risk interval from influenza to the onset of immune thrombocytopenia, considering influenza episode more than 30 days apart separately

A: Nested case–control study

B: Self-controlled case series

**Table S7.** Post hoc analysis: Risk interval from influenza to the onset of immune thrombocytopenia with narrow risk intervals

A: Nested case–control study

B: Self-controlled case series

**Table S1.** Definitions of International Classification of Diseases 10th Revision codes, Japanese procedure codes, and Anatomical Therapeutic Chemical codes

| Categories | Codes |
| --- | --- |
| **Outcomes** |  |
| Diagnostic code |  |
| ・Immune thrombocytopenia | D693, D694 |
| ・Excluded diseases | D688, D695, D696, M311, R768 |
| Procedure code |  |
| ・Complete blood count test | 160191510, 160008010, 160008210, 160061810, 160095970 |
| **Exposures** |  |
| Diagnostic code |  |
| ・Influenza | J09, J10, J11 |
| Procedure Code |  |
| ・Influenza | 160169450 (Rapid antigen test for Influenza) 160198010 (Polymerase Chain Reaction test for Influenza) |
| ATC Code |  |
| ・Anti-influenza drug | J05AH01, J05AH02, J05AH04, J05AX25 |
| **Pediatric Complex Chronic Conditions version 3 (Procedure code)** | |
| ・Neurologic | F71, F72, F73, E75, F842, G11, G12, G310, G318, G319, G328, G909, G910, G911, G913, G914, G918, G919, G938, G939, G94, G951, G958, Q851, G80, G400, G401, G402, G403, G404, G408, G409, G361, G368, G369, G37, G81, G825, G835, G839, G931, G935, M414, M415, R403, I63, G71, G72, G10, G20, G21, G23, G240, G241, G242, G248, G255, G258, G259, G26, G803 |
| ・Cardiovascular | Q20, Q212, Q213, Q214, Q218, Q219, Q22, Q23, Q24, Q251, Q252, Q253, Q254, Q255, Q256, Q257, Q258, Q259, Q26, Q282, Q283, Q288, Q289, I340, I342, I348, I349, I36, I37, I42, I43, I51.5, I44, I45, I47, I48, I490, I491, I493, I494, I495, I498, I499, R001, I27, I502, I503, I504, I508, I509, I517, I518, I630, I631, I632, Z951, T862, T863, Z941, Z943 |
| ・Respiratory | Q30, Q31, Q32, Q33, Q34, G473, I270, I278, J841, J842, J848, J849, J961, J962, Z902, E84, T863, T868, Z942, Z943 |
| ・Renal and Urologic | Q60, Q61, Q62, Q63, Q64, Z877, N18, Z905, Z906, G834, N31, T861, Z940 |
| ・Gastrointestinal | Q390, Q391, Q392, Q393, Q394, Q41, Q42, Q431, Q432, Q433, Q434, Q435, Q436, Q437, Q438, Q439, Q44, Q45, Z877, K73, K74, K754, K760, K761, K762, K763, K765, K768, K50, K51, I820, K551, K562, K593, Z903, Z904, Z980, T864, T868, Z944, Z948 |
| ・Hematologic or Immunologic | D55, D56, D57, D58, D60, D61, D71, D720, D80, D81, D82, D83, D84, D894, D898, D899, M303, M359, D66, D682, D694, D700, D704, D76, D86, B20, Z21, M300, M301, M302, M308, M310, M311, M313, M314, M315, M316, M317, M321, M328, M329, M33, M34 |
| ・Metabolic | E70, E71, E72, E74, E75, E77, E78, E881, E888, E76, E85, M041, D841, E791, E792, E798, E799, E803, E804, E805, E806, E807, E830, E831, E833, E834, E880, E882, E883, E884, E889, H498, E00, E030, E031, E222, E230, E232, E233, E237, E240, E242, E243, E248, E249, E25, E268, Z794 |
| ・Other Congenital or Genetic Defect | Q90, Q91, Q92, Q93, Q950, Q952, Q953, Q955, Q958, Q959, Q96, Q97, Q98, Q990, Q991, Q998, Q999, E343, E349, M410, M433, M965, Q720, Q721, Q722, Q75, Q760, Q761, Q762, Q764, Q765. Q766, Q767, Q77, Q780, Q781, Q782, Q783, Q784, Q786, Q788, Q789, K449, Q790, Q791, Q792, Q793, Q794, Q795, Q799, Z877, Q81, Q870, Q871, Q872, Q873, Q874, Q878, Q897, Q898, Q899, Q992 |
| ・Malignancy | C00–C96, D00-D09, D37–D49, Q850, Q858, T860, T865, Z948 |
| ・Premature and Neonatal | P050, P051, P052, P059, P070, P072, P100, P101, P104, P108, P109, P522, P524, P255, P256, P528, P259, P115, P25, P27, P916, P111, P112, P119, P350, P351, P354, P560, P569, P57, P613, P614, P773, P832, P912, Z876 |
| ・Device and Technology usage | T850, T851, T857, T858, Z454, Z968, Z982, T820, T821, T822, T823, T824, T82.5, T826, T827, T828, T829, Z451, Z450, Z950, Z952, Z953, Z954, Z958, J950, J958, Z430, Z930, Z990, Z991, T857, Z435, Z436, Z49, Z911, Z935, Z936, Z992, K942, Z431, Z432, Z433, Z434, Z465, Z931, Z932, Z933, Z934, Z468, Z964, T870, T871, T872, Y831, Y833, Z998 |
| ・Transplant | T860, T861, T862, T863, T864, T865, T868, T869, Z940, Z941, Z942, Z943, Z944, Z948 |

**Table S2.** Sensitivity analysis 1: Risk interval from influenza to hospitalization for immune thrombocytopenia

A: Nested case–control study

| Risk interval (days) | Controls | Cases | Adjusted odds ratio |
| --- | --- | --- | --- |
|  | (n = 9,043) | (n = 915) | (95% confidence interval) |
| 1–7 | 13 | 4 | 3.57 (1.12–11.36) |
| 8–14 | 19 | 3 | 1.83 (0.54–6.21) |
| 15–21 | 12 | 2 | 1.66 (0.37–7.42) |
| 22–28 | 16 | 2 | 1.27 (0.29–5.61) |
| No infection within 28 days | 8,983 | 904 | 1 [Reference] |

B: Self-controlled case series

| Risk interval (days) | Events (n) | Person-days | Incidence rate ratio  (95% confidence interval) |
| --- | --- | --- | --- |
| Baseline period | 412 | 1,317,758 | 1 [Reference] |
| Pre-exposure period | 0 | 10,147 | – |
| Day 0 | 4 | 755 | 13.84 (4.94–38.77) |
| 1–7 | 4 | 5,278 | 2.04 (0.72–5.75) |
| 8–14 | 3 | 5,271 | 1.67 (0.52–5.31) |
| 15–21 | 2 | 5,220 | 1.21 (0.30–4.93) |
| 22–28 | 2 | 5,126 | 1.24 (0.30–5.07) |

A: Conditional logistic regression was performed to estimate the odds ratios comparing influenza episodes among cases and controls, adjusting for each of the following categories: age, sex, time of registration in the database, each category of the Pediatric Complex Chronic Condition System version 3, number of hospital visits, number of medicines prescribed, and history of admission to the pediatric department.

B: The incidence rate ratio was estimated using a conditional Poisson regression and adjusted for age band and seasonal effects.

**Table S3.** Sensitivity analysis 1: Risk interval from influenza to the administration of immune thrombocytopenia treatment

A: Nested case–control study

| Risk interval (days) | Controls | Cases | Adjusted odds ratio |
| --- | --- | --- | --- |
|  | (n = 8,034) | (n = 813) | (95% confidence interval) |
| 1–7 | 10 | 4 | 5.00 (1.48–16.91) |
| 8–14 | 16 | 4 | 2.81 (0.94–8.46) |
| 15–21 | 11 | 2 | 1.89 (0.42–8.57) |
| 22–28 | 14 | 2 | 1.52 (0.34–6.87) |
| No infection within 28 days | 7,983 | 801 | 1 [Reference] |

B: Self-controlled case series

| Risk interval (days) | Events (n) | Person-days | Incidence rate ratio  (95% confidence interval) |
| --- | --- | --- | --- |
| Baseline period | 367 | 1,170,270 | 1 [Reference] |
| Pre-exposure period | 0 | 8,931 | – |
| Day 0 | 3 | 664 | 11.46 (3.49–37.70) |
| 1–7 | 4 | 4,643 | 2.18 (0.77–6.22) |
| 8–14 | 4 | 4,634 | 2.42 (0.88–6.69) |
| 15–21 | 2 | 4,577 | 1.32 (0.32–5.38) |
| 22–28 | 2 | 4,509 | 1.34 (0.32–5.52) |

A: Conditional logistic regression was performed to estimate the odds ratios comparing influenza episodes among cases and controls, adjusting for each of the following categories: age, sex, time of registration in the database, each category of the Pediatric Complex Chronic Condition System version 3, number of hospital visits, number of medicines prescribed, and history of admission to the pediatric department.

B: The incidence rate ratio was estimated using a conditional Poisson regression and adjusted for age band and seasonal effects.

**Table S4.** Sensitivity analysis 2: Risk interval from influenza treated with anti-influenza medication to the onset of immune thrombocytopenia

A: Nested case–control study

| Risk interval (days) | Controls | Cases | Adjusted odds ratio |
| --- | --- | --- | --- |
|  | (n = 12,878) | (n = 1,306) | (95% confidence interval) |
| 1–7 | 26 | 5 | 2.21 (0.82–5.95) |
| 8–14 | 28 | 5 | 2.08 (0.79–5.48) |
| 15–21 | 23 | 1 | 0.32 (0.04–2.48) |
| 22–28 | 20 | 3 | 1.39 (0.41–4.73) |
| No infection within 28 days | 12,781 | 1,292 | 1 [Reference] |

B: Self-controlled case series

| Risk interval (days) | Events (n) | Person-days | Incidence rate ratio  (95% confidence interval) |
| --- | --- | --- | --- |
| Baseline period | 629 | 1,952,204 | 1 [Reference] |
| Pre-exposure period | 3 | 14,525 | 0.38 (0.12–1.25) |
| Day 0 | 0 | 1,072 | – |
| 1–7 | 5 | 7,494 | 1.63 (0.64–4.16) |
| 8–14 | 5 | 7,490 | 1.72 (0.69–4.27) |
| 15–21 | 1 | 7,402 | 0.39 (0.05–2.79) |
| 22–28 | 3 | 7,287 | 1.19 (0.37–3.78) |

A: Conditional logistic regression was performed to estimate the odds ratios comparing influenza episodes among cases and controls, adjusting for each of the following categories: age, sex, time of registration in the database, each category of the Pediatric Complex Chronic Condition System version 3, number of hospital visits, number of medicines prescribed, and history of admission to the pediatric department.

B: The incidence rate ratio was estimated using a conditional Poisson regression and adjusted for age band and seasonal effects.

**Table S5.** Sensitivity analysis: 3 Risk interval from influenza to the onset of immune thrombocytopenia, considering influenza episodes more than 7 days apart separately

A: Nested case–control study

| Risk interval (days) | Controls | Cases | Adjusted odds ratio |
| --- | --- | --- | --- |
|  | (n = 12,878) | (n = 1,306) | (95% confidence interval) |
| 1–7 | 30 | 9 | 3.50 (1.59–7.69) |
| 8–14 | 30 | 5 | 2.01 (0.76–5.27) |
| 15–21 | 26 | 2 | 0.63 (0.14–2.71) |
| 22–28 | 24 | 3 | 1.09 (0.32–3.74) |
| No infection within 28 days | 12,768 | 1,287 | 1 [Reference] |

B: Self-controlled case series

| Risk interval (days) | Events (n) | Person-days | Incidence rate ratio  (95% confidence interval) |
| --- | --- | --- | --- |
| Baseline period | 650 | 2,032,765 | 1 [Reference] |
| Pre-exposure period | 3 | 15,970 | 0.38 (0.12–1.23) |
| Day 0 | 4 | 1,206 | 8.19 (2.98–22.50) |
| 1–7 | 9 | 8,431 | 2.71 (1.34–5.46) |
| 8–14 | 5 | 8,355 | 1.61 (0.65–3.96) |
| 15–21 | 2 | 8,203 | 0.72 (0.18–2.91) |
| 22–28 | 3 | 8,059 | 1.11 (0.35–3.50) |

A: Conditional logistic regression was performed to estimate the odds ratios comparing influenza episodes among cases and controls, adjusting for each of the following categories: age, sex, time of registration in the database, each category of the Pediatric Complex Chronic Condition System version 3, number of hospital visits, number of medicines prescribed, and history of admission to the pediatric department.

B: The incidence rate ratio was estimated using a conditional Poisson regression and adjusted for age band and seasonal effects.

**Table S6.** Sensitivity analysis 3: Risk interval from influenza to the onset of immune thrombocytopenia, considering influenza episode more than 30 days apart separately

A: Nested case–control study

| Risk interval (days) | Controls | Cases | Adjusted odds ratio |
| --- | --- | --- | --- |
|  | (n = 12,878) | (n = 1,306) | (95% confidence interval) |
| 1–7 | 29 | 8 | 3.21 (1.41–7.28) |
| 8–14 | 30 | 6 | 2.38 (0.97–5.84) |
| 15–21 | 26 | 2 | 0.63 (0.14–2.72) |
| 22–28 | 25 | 3 | 1.06 (0.31–3.62) |
| No infection within 28 days | 12,768 | 1,287 | 1 [Reference] |

B: Self-controlled case series

| Risk interval (days) | Events (n) | Person-days | Incidence rate ratio  (95% confidence interval) |
| --- | --- | --- | --- |
| Baseline period | 650 | 2,033,753 | 1 [Reference] |
| Pre-exposure period | 3 | 15,969 | 0.38 (0.12–1.24) |
| Day 0 | 4 | 1,150 | 8.48 (3.07–23.42) |
| 1–7 | 8 | 8,039 | 2.50 (1.18–5.26) |
| 8–14 | 6 | 8,029 | 1.99 (0.87–4.55) |
| 15–21 | 2 | 8,029 | 0.73 (0.18–2.96) |
| 22–28 | 3 | 8,020 | 1.11 (0.35–3.53) |

A: Conditional logistic regression was performed to estimate the odds ratios comparing influenza episodes between cases and controls, adjusting for each of the following categories: age, sex, time of registration in the database, each category of the Pediatric Complex Chronic Condition System version 3, number of hospital visits, number of medicines prescribed, and history of admission to the pediatric department.

B: The incidence rate ratio was estimated using a conditional Poisson regression and adjusted for age band and seasonal effects.

**Table S7.** Post-hoc analysis: Risk interval from influenza to the onset of immune thrombocytopenia with narrow risk intervals.

A: Nested case–control study

| Risk interval (days) | Controls | Cases | Adjusted odds ratio |
| --- | --- | --- | --- |
|  | (n = 12,878) | (n = 1,306) | (95% confidence interval) |
| 1–3 | 9 | 2 | 2.74 (0.57–13.11) |
| 4–7 | 21 | 6 | 3.35 (1.31–8.61) |
| 8–10 | 7 | 4 | 6.38 (1.84–22.07) |
| 11–14 | 23 | 2 | 1.07 (0.25-4.59) |
| 15–21 | 26 | 2 | 0.63 (0.15–2.75) |
| 22–28 | 24 | 3 | 1.09 (0.32–3.72) |
| No infection within 28 days | 12,768 | 1,287 | 1 [Reference] |

B: Self-controlled case series

| Risk interval (days) | Events (n) | Person-days | Incidence rate ratio  (95% confidence interval) |
| --- | --- | --- | --- |
| Baseline period | 650 | 2,032,969 | 1 [Reference] |
| Pre-exposure period | 3 | 15,970 | 0.38 (0.12–1.23) |
| Day 0 | 4 | 1,188 | 8.28 (3.00–22.85) |
| 1–3 | 2 | 3,562 | 1.42 (0.34–5.85) |
| 4–7 | 6 | 4,743 | 3.25 (1.40–7.54) |
| 8–10 | 4 | 3,555 | 3.01 (1.11–8.20) |
| 11–14 | 2 | 4,740 | 1.22 (0.30–4.96) |
| 15–21 | 2 | 8,203 | 0.72 (0.18–2.91) |
| 22–28 | 3 | 8,059 | 1.11 (0.35–3.50) |

A: Conditional logistic regression was performed to estimate the odds ratios comparing influenza episodes among cases and controls, adjusting for each of the following categories: age, sex, time of registration in the database, each category of the Pediatric Complex Chronic Condition System version 3, number of hospital visits, number of medicines prescribed, and history of admission to the pediatric department.

B: The incidence rate ratio was estimated using a conditional Poisson regression and adjusted for age band and seasonal effects.
